# The Effect of Probiotic Yogurt on Glycemic Control in Type 2 Diabetes or Obesity: A Meta-Analysis of Nine Randomized Controlled Trials

**DOI:** 10.3390/nu11030671

**Published:** 2019-03-20

**Authors:** Elena Barengolts, Emily Daviau Smith, Sirimon Reutrakul, Livia Tonucci, Thunyarat Anothaisintawee

**Affiliations:** 1 Division of Endocrinology, Diabetes, and Metabolism, Department of Medicine, University of Illinois, Chicago, IL 60612, USA; edavia2@uic.edu (E.D.S.); sreutrak@uic.edu (S.R.); 2 Section of Endocrinology, Department of Medicine, Jesse Brown VA Medical Center, Chicago, IL 60612, USA; 3 Department of Nutrition, INTA University, Sobral 62050-130, Brazil; livia_bordalo@hotmail.com; 4 Department of Family Medicine, Faculty of Medicine Ramathibodi Hospital, Mahidol University, Bangkok 10400, Thailand; thunyarat.ano@mahidol.ac.th

**Keywords:** probiotic yogurt, conventional yogurt, kefir, hemoglobin A1c, body mass index, glucose, HOMA-IR, type 2 diabetes, obesity, a meta-analysis, randomized controlled trials

## Abstract

Probiotic yogurt is suggested as a nutritional approach in type 2 diabetes (T2D) and obesity. We performed a systematic review and meta-analysis of randomized controlled trials (RCTs) evaluating the effects of probiotic yogurt on glycemic outcomes in T2D or obesity. The databases used to search for RCTs included Medline and Scopus. The RCTs were eligible if outcomes included selected glycemic markers. In nine eligible trials, 237 and 235 subjects were in treatment (probiotic yogurt) and control (mostly conventional yogurt) groups, respectively. There was no significant difference for pooled unstandardized mean difference (USMD) hemoglobin A1c (HbA1c) by probiotic yogurt compared with the control in T2D (USMD: −0.366; 95% CI: −0.755, 0.024, *p* = 0.066) and obesity (USMD: 0.116, 95% CI: −0.007, 0.238, *p* = 0.065). Similarly, there were no effects of probiotic yogurt on fasting blood glucose, fasting insulin, or insulin resistance (estimated by homeostatic model assessment of insulin resistance (HOMA-IR)) in either T2D or obesity. In conclusion, the present meta-analysis has not demonstrated the benefits of consuming probiotic compared with conventional yogurt for improving glucose control in patients with diabetes or obesity. Larger trials are needed to verify the benefits of probiotic and/or conventional yogurt or other probiotic fermented milk (e.g., kefir) on glycemic markers in patients with diabetes and obesity.

## 1. Introduction

The metabolic disorders type 2 diabetes (T2D) and obesity are highly prevalent in the US. There are approximately 30 million individuals with T2D and nearly 90 million with prediabetes [1], while about 100 million individuals are obese and an additional 80 million are overweight [2]. Diabetes and obesity are also highly prevalent worldwide. There are multiple emerging treatments for T2D and obesity, but the management of both conditions remains challenging to physicians and burdensome to society [3,4,5,6]. The co-risks of T2D and obesity are genetics and lifestyle [4,6,7,8]. The individual genetic code is usually considered as nonmodifiable; however, the human gut microbiome, i.e., the genetic code of gut microbacteria, could possibly be modified by lifestyle. Lifestyle, particularly nutrition incorporating probiotics, could offer a novel approach to metabolic disorders.

Probiotics are defined as microorganisms that are accepted as beneficial for humans. The view is attributed to Nobel laureate Élie Metchnikoff, who suggested in 1907 “to replace the harmful microbes by useful microbes” [9,10]. The World Health Organization’s definition of probiotics is “live microorganisms which, when administered in adequate amounts, confer a health benefit on the host” [11]. Several recent reviews and meta-analyses, including meta-analyses of randomized controlled trials (RCTs), have suggested the usefulness of probiotics for glycemic benefits in T2D and obesity [12,13,14]. These meta-analyses, however, predominantly involved probiotic capsules. Probiotic bacteria are also used for the production of various foods, including probiotic yogurt [10,15,16]. Probiotic yogurt is expected to contain multiple bacteria, including *Lactobacillus acidophilus*, *Bifidobacterium bifidum*, *Bifidobacterium lactis*, *Lactobacillus casei*, and/or *Lactobacillus rhamnosus* among others [10,15]. These bacteria, which are suggested to be beneficial for human health, are present in probiotic yogurt in addition to *Streptococcus thermophilus* and *Lactobacillus Bulgaricus*, which are the most frequent microorganisms used to produce conventional yogurt [16]. Yogurt, including probiotic yogurt, is a source of protein (8.5 g per cup) that contains all nine of the essential amino acids [17]. Yogurt and fermented milk have proven association with improved overall health and decreased all-cause mortality [18,19,20,21,22,23], although there have not been any published studies dedicated to the association of probiotic supplements with mortality. Based on animal and human research, yogurt is recommended as part of healthy nutrition (and not supplements) as an essential part of managing T2D and T2D complications [7]. Probiotic supplements are expensive, and the manufacturing is not monitored by the US Food and Drug Administration (FDA), making content undependable [10]. These differences between probiotic yogurt and supplements make probiotic yogurt a preferred approach in patients with T2D if glycemic benefits were to be supported by clinical trials.

There have been several randomized controlled trials evaluating the effects of probiotic yogurt on glycemic outcomes in patients with T2D, and these trials have produced contradictory results [24,25,26,27,28,29,30]. There are, however, no meta-analyses of the RCTs dedicated to the effects of probiotic yogurt on glycemic outcomes in T2D or obesity. The present review addresses this gap of knowledge. In the present study, we performed a meta-analysis of RCTs that utilized probiotic yogurt as a main intervention in participants with T2D or obesity.

## 2. Methods

Data sources and searches: The relevant studies were searched on Medline and Scopus databases starting from January 1995 through to November 2017. Additional relevant studies were identified from the list of reference from included publications. Search terms included “dairy”, “milk”, “probiotic”, “fermented dairy”, “fermented milk”, “probiotic fermented milk”, “yogurt”, “conventional yogurt”, and “probiotic yogurt”. It also included various names for fermented milk, i.e., “buttermilk”, “kefir”, “lassi”, “doogh”, “dahi”, “amasi”, “filmjolk”, and “chal”. The strategy in Scopus was to include all names as “TITLE-ABS-KEY” and to combine each of those names with “type 2 diabetes”. The strategy in Medline was to use the same terms in “mesh” and sort by relevance.

Study selection: Two reviewers (E.B. and E.D.S.) independently selected the studies. The study selection process was based on preferred reporting items for systematic reviews and meta-analyses (PRISMA) [31]. Meetings and discussions helped to resolve disagreements between reviewers, and consultations with other authors were sought as needed. The published studies were deemed eligible if they met all of the following criteria: English language; randomized controlled trial (either parallel groups or cross-over); population of adults (age older than 18 years) with type 2 diabetes or at risk for T2D (healthy populations were excluded); duration of at least 2 weeks; probiotic dairy product used as an intervention (other probiotic products, e.g., bread and capsules, were excluded); and any dairy control (e.g., conventional yogurt, milk, etc.) or dietary advice (juice as control was excluded). To be eligible, the outcomes needed to include at least one of the selected glycemic markers (see below), and the reported data needed to be sufficient for extraction.

Data extraction: Two reviewers autonomously collected the data using a standardized data record form. Discrepancies were resolved by consultations and consensus with other authors. Multiple attempts were made to contact researchers of the publications with missing data.

Trial characteristics: The study design included determining whether it was randomized, controlled, double- or single-blind, or cross-over as well as the duration. Intervention included detailed description of fermented dairy product, including bacterial cultures used for fermentation. Only probiotic fermented dairy products (from cow or goat milk) were included, while other probiotic-containing products, e.g., bread or capsules, were excluded. Comparator controls were included if these were nonfermented, fermented, or artificially acidified milk or dietary advice, but the trial was excluded if juice was used as a comparator. Participant characteristics included age and gender. Only patients with T2D or at high risk for T2D (i.e., obesity) were included; if participants were characterized as “healthy”, the trial was excluded from analysis.

Outcomes of interest: The outcomes of interest were glycemic markers, i.e., hemoglobin A1c (HbA1c), fasting blood glucose (FBG), fasting insulin, and insulin resistance (IR) estimated by the homeostatic model assessment of insulin resistance (HOMA-IR). The trials were included if the data were available at least for one outcome of interest.

Risk of bias assessment: The risk of bias assessment was performed by the authors according to the criteria detailed in the Cochrane Collaboration’s tool [32]. Seven domains were assessed: random sequence generation, allocation concealment, blinding of participants and personnel, blinding of outcome assessment, incomplete outcome data, selective reporting, and other bias. The risk of bias in each domain was classified as low, high, or unclear risk [32].

Data synthesis and analysis: The mean difference of HbA1c, fasting blood glucose, and insulin level between probiotic yogurt and control groups of each study were estimated and were pooled using unstandardized mean difference (USMD). Heterogeneity between studies was measured by the *Q*-test and the I^2^ statistic (degree of heterogeneity). Heterogeneity between studies was presented; if the *p*-value from the *Q*-test was less than 0.1 and/or the I2 was greater than 25%. USDM was estimated using fix-effect model if there was no heterogeneity between studies; otherwise, the random effect model was applied.

Publication bias was assessed only for HbA1c and FBS in T2D because the number of included studies for these outcomes was greater than two. Publication bias was assessed using funnel plot. If there was asymmetry from the funnel plot, contour-enhanced funnel plot was performed to assess whether asymmetry had resulted from the small-study effect or the heterogeneity between studies.

All statistical analyses were performed using STATA version 15. A *p*-value < 0.05 was considered as statistically significant for all tests except for the heterogeneity test in which a *p*-value < 0.10 was used.

## 3. Results

### 3.1. Description of Included Trials

**Figure 1:**
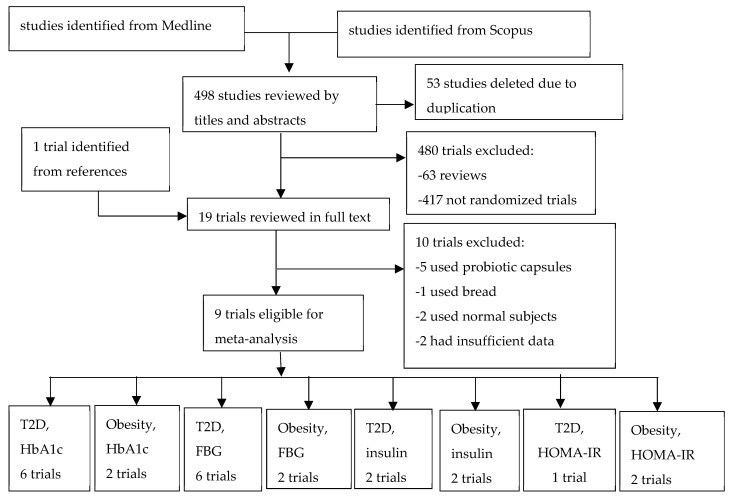
Flow chart of trial selection.

A total of 551 studies were identified in Scopus and Medline, and one study was added from the references. Nine studies met the inclusion criteria and were included in the meta-analysis (Figure 1). The characteristics of these studies are described in Table 1. Of the nine trials included in the meta-analysis, seven were conducted in subjects with T2D [24,25,26,27,28,29,30] and two were conducted in subjects with obesity [33,34]. A total of 237 and 235 were in treatment and control groups, respectively. Two trials included exclusively women [28,34], and one trial included exclusively men [26]. A majority of subjects were young or middle aged. Diabetes control overall was reasonable, with HbA1c below 9%. Obese subjects had HbA1c in normal or prediabetes range.

The attributes of the studies were evaluated. All studies had good descriptions of the design and interventions. All studies used randomized controlled parallel group design; seven were double blind, one was single blind, and one was open-label. The intervention in all the trials was probiotic fermented milk, with eight using cow milk and one using goat milk. Among the cow-milk yogurts, one study used a symbiotic shake and one used kefir. The composition of fermentation cultures was provided in all but one trial. The majority of the trials (*n* = 7) used blends of various probiotic genera, including lactobacilli and bifidobacteria. The control included conventional yogurt (*n* = 5 trials), conventional milk (*n* = 2), artificially acidified milk (*n* = 1), or dietary advice (*n* = 1). Baseline level of glycemic measures were similar between control and treatment groups but for HbA1c in one trial [28]. All T2D studies described the use of T2D medications. The funding did not involve industry in all but one trial [25]. Attrition was low in all studies, but a one trial had more than 50% attrition [28]. Two trials in obese subjects comprised overweight and obese participants without T2D, including a study on women during a weight-loss program [34] and a study on men and postmenopausal women without and with prediabetes [33]. A study in patients with T2D that had sufficient data only for HbA1c was included in the meta-analysis of HbA1c but not included in the analysis of FBG, insulin, and HOMA-IR due to insufficient data for these parameters [26].

### 3.2. Risk of Bias Assessment

**Figure 2:**
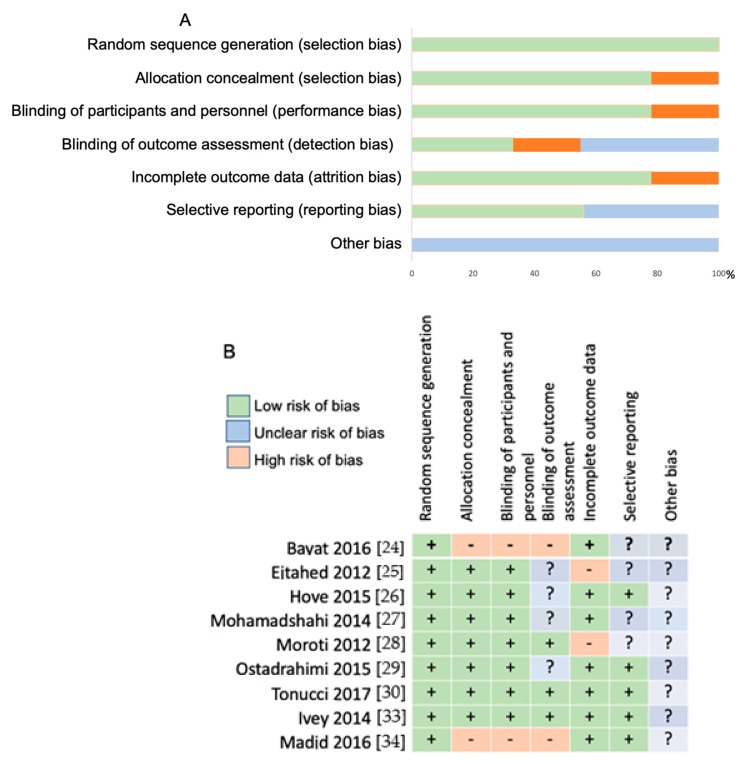
Risk of (**A**) bias graph and (**B**) risk of bias summary in nine trials.

Figure 2 summarizes the authors’ assessment of bias. Included in this review were selection bias (sequence generation and allocation concealment), blinding for performance and detection bias, incomplete outcome data for attrition bias, selective reporting for reporting bias, and other potential bias (Figure 2). The majority of trials were assessed as presenting with either low or unclear risk of bias.

**Figure 3:**
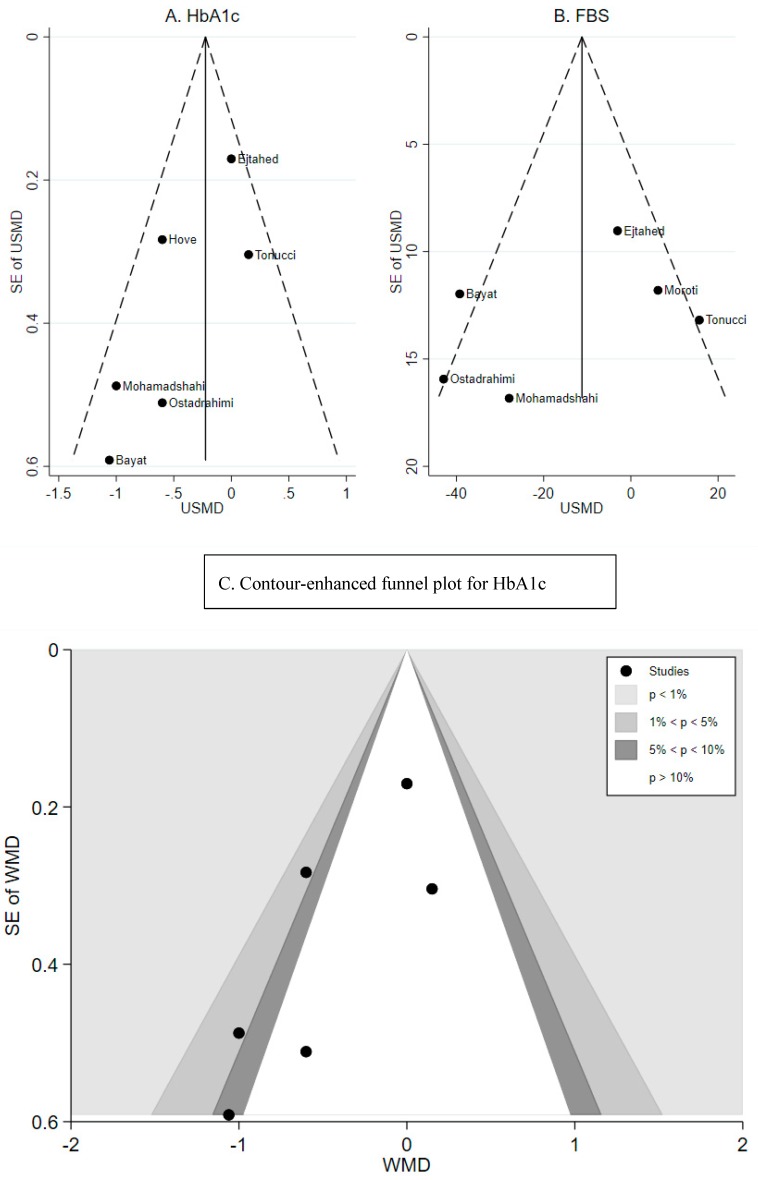
Risk of publication bias. The funnel plots (**A**) HbA1c, (**B**) fasting blood glucose, and (**C**) the contour-enhanced funnel plot for HbA1c.

Publication bias: Publication bias was assessed by the funnel plot where the X-axis represents the mean treatment effect and Y-axis represents the inverse standard error. The vertical line represents the pooled effect size. If there is no publication bias, the studies with high precision will be plotted near the pooled effect size, and the studies with low precision will be spread symmetrically on both sides of the pooled effect size. The publication bias will be assumed if the funnel plot is asymmetrical, i.e., some small studies are missing in the area of negative results. For this study, the funnel plots showed asymmetry for only HbA1c outcome (Figure 3A) and not for FBG (Figure 3B). The contour-enhanced funnel plot was further performed, and it suggested that there was missing study in the nonsignificant area (Figure 3C). Therefore, the asymmetrical funnel plot of HbA1c outcome likely resulted from publication bias rather than heterogeneity between studies. Table 2 shows the baseline and final glycemic measures as well as published statistical differences between treatment and control for all trials. A majority of studies were adjusted for baseline values, duration of diabetes, and medication use.

### 3.3. Effect of Probiotic Yogurt on HbA1c

**Figure 4:**
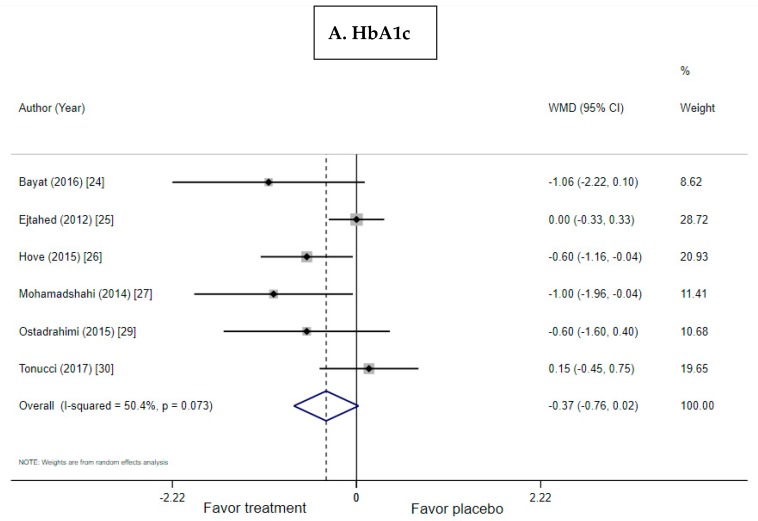

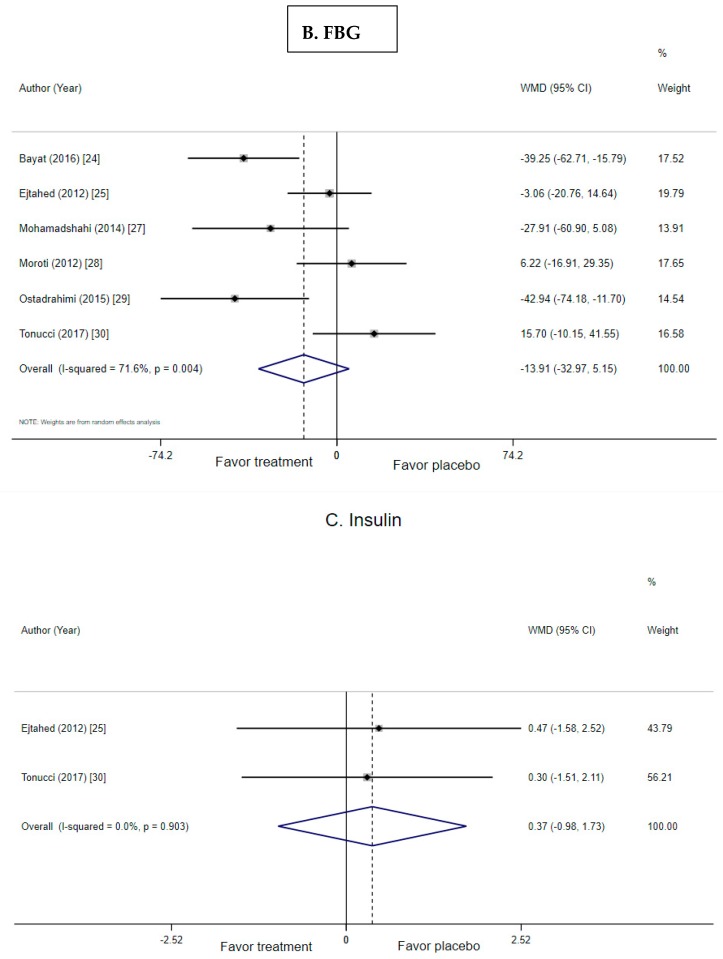
Forest plots for the effect of probiotic yogurt in T2D on (**A**) HbA1c, (**B**) FBG, and (**C**) insulin compared with the control in pooled analysis.

**Figure 5:**
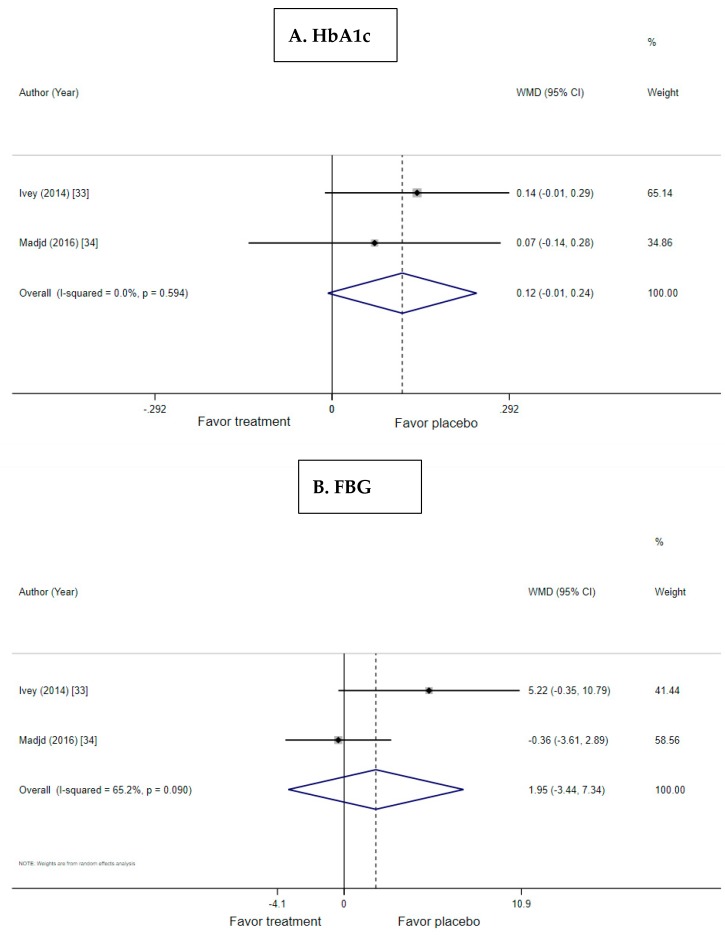

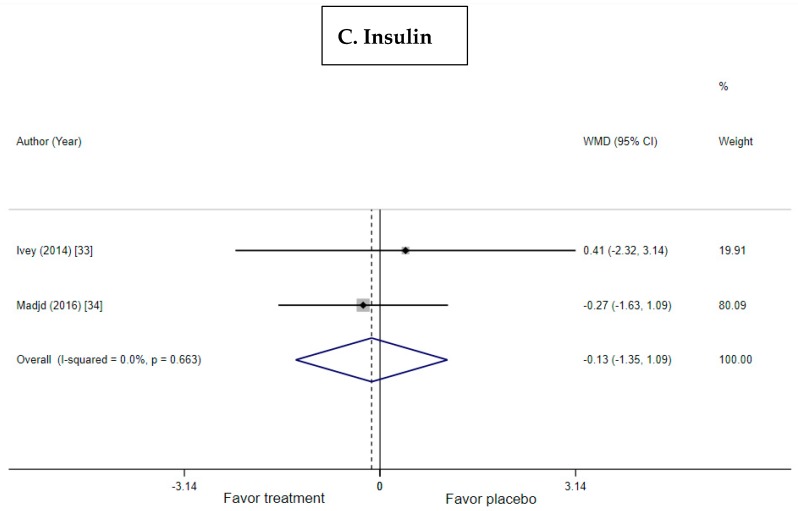
Forest plots for the effect of probiotic yogurt in obesity on (**A**) HbA1c, (**B**) FBG, and (**C**) insulin compared with the control in pooled analysis. T2D insulin data were only available in a single trial precluding the meta-analysis.

The meta-analysis included six trials of T2D and two trials of obesity (Figure 4A and Figure 5A). There was a nonstatistically significant trend for USMD toward reduction of HbA1c by probiotic yogurt compared with the control when used by patients with T2D (USMD: −0.366; 95% CI: −0.755, 0.024, *p* = 0.066; I^2^ = 50%, *p* = 0.073 for heterogeneity). Similarly, there was a nonsignificant trend for improving HbA1c in obese subjects without T2D (USMD: 0.116, 95% CI: −0.007, 0.238, *p* = 0.065; I^2^ = 0%, *p* = 0.594 for heterogeneity).

### 3.4. Effect of Probiotic Yogurt on Fasting Blood Glucose

The meta-analysis included six trials of T2D and two trials of obesity. Published differences in final FBG between treatment and control were significant in four of the six trials in subjects with T2D (Table 2). The meta-analysis, however, showed that there was no difference in FBG in subjects on probiotic yogurt compared with the control (USMD: −13.91; 95% CI: −32.97, 5.51, *p* = 0.153) (Figure 4B). There was also no effect of probiotic yogurt on FBG in obese participants without T2D (Figure 5B).

### 3.5. Effect of Probiotic Yogurt on Insulin and HOMA-IR

The data for insulin were available from two studies of T2D and two studies of obesity (Figure 4C and Figure 5C). There was no effect of probiotic yogurt on insulin in either T2D or obesity. There was no effect of probiotic yogurt on HOMA-IR in the meta-analysis of the two available trials in obesity (USMD: −0.002, 95% CI: −0.302, 0.297, *p* = 0.988).

## 4. Discussion

This study systematically reviewed and meta-analyzed nine randomized controlled trials (472 participants) to clarify the effects of probiotic yogurt on glycemic markers in diabetes and obesity. Overall, it appears that probiotic yogurt provided no significant improvement compared with the control in glycemic markers including HbA1c, fasting insulin and glucose, and insulin resistance measured by HOMA-IR.

To our knowledge, this is the first meta-analysis that is dedicated specifically to the glycemic effects of probiotic yogurt and has excluded probiotic capsules in patients with established T2D or at high risk for T2D (i.e., obesity/overweight). The results varied from other meta-analyses of RCTs, and several factors could explain this disagreement. Previous meta-analyses showing that probiotics improved glycemic outcomes [13,14,35,36,37] encompassed a variety of probiotic products, including capsules [38,39,40,41,42,43,44] and bread [45] in addition to yogurt. Participants included in previous meta-analyses also varied and involved T2D, gestational diabetes, metabolic syndrome, obesity, and healthy subjects. In addition, the use of conventional yogurt as control [25,27,28,29,34] might have influenced results. Conventional yogurt might have provided glycemic improvement obviating benefits of probiotic yogurt. Differences in the qualitative and quantitative amount of active living bacteria in probiotic and conventional yogurt can be minimal, which explains the lack of beneficial glycemic effects of probiotic yogurt. The FDA standards require that conventional yogurt cultures should contain *Lactobacillus bulgaricus* and *Streptococcus thermophilus*, but the FDA has no requirements on the number (i.e., colony-forming unit (CFU)) of these bacteria [46]. There have been no RCTs comparing conventional yogurt to milk or dietary advice to assess whether yogurt per se could improve glycemic control in T2D. Prospective cohort studies [47,48,49,50,51,52] and their meta-analyses [48,53,54,55] have shown that yogurt is associated with lower risk of developing T2D. There are, however, no prospective studies evaluating the effects of yogurt on the outcomes of macro- and microvascular complications in patients with T2D.

A single trial included in our meta-analysis compared kefir with conventional yogurt in patients with T2D [29]. The trial was small (*n* = 60) and of short duration (8 weeks); yet, it showed a reduction in HbA1c and FBG. Kefir, which is similar to conventional or probiotic yogurt, belongs to fermented milk products. Food standards define yogurt as a form of fermented milk that contains symbiotic cultures of *Streptococcus thermophilus* subsp. *salivarius* and *Lactobacillus delbrueckii* subsp. *bulgaricus*. Probiotic yogurt has the additional strains of probiotic bacteria, such as *Bifidobacterium lactis* or *Lactobacillus acidophilus* [10,11,15]. Kefir, which is similar to yogurt, contains live bacterial cultures and also has yeasts, fungi, and kefiran, a water-soluble polysaccharide [56]. Kefir is proclaimed to contain more than 600 unique peptides and to offer multiple health benefits, such as improved lipid profile, angiotensin-converting enzyme inhibition, tumor suppression, and antimicrobial and anti-inflammatory activity [56]. There is, however, insufficient evidence from RCTs to substantiate these claims [29,57,58,59].

Multiple previous preclinical and clinical studies and reviews have provided data and suggested mechanistic insights explaining glycemic and other health benefits of yogurt and probiotics. These mechanisms involve all organs and systems from the brain to the gut and the immune system and are mediated by pathways related to pancreatic beta-cell preservation [60], hepatic glucose production [61], incretin hormones synthesis and secretion [62], peroxisome proliferator-activated receptors [63], fatty acid oxidation [64], endotoxin [65], innate immunity [66], and appetite suppression [67] among others. In addition to the benefits of yogurt, kefir could offer a unique antidiabetic potential. Kefir has been shown to improve lipid profile, insulin resistance, fasting glucose, and fasting insulin; reduce weight gain; decrease products of lipid oxidation and proinflammatory cytokine expression (IL-1β, TNFα, IL-6); and to increase anti-inflammatory cytokine expression (IL-10) in diet-induced animal rodent model of diabetes [68,69]. In insulin-responsive muscle cells, kefir has been shown to enhance glucose uptake and reduce reactive oxygen species (ROS), possibly through activation of PI 3-kinases [70]. Kefir has also been shown to decrease oxidative stress in streptozotocin-induced diabetes rat model [71] and in rat model of myocardial infarction [72]. Kefir was found to decrease physical fatigue and improve exercise performance in nondiabetic mice. The suggested mechanism was that animals were able to utilize lactate as an energy source and decrease accumulation of blood ammonia [73]. *Lactobacillus plantarum* CIDCA 8327 strain, which is uniquely found in kefir grains [74], has been suggested as a possible producer of kefir exopolysaccharides, including dextrans and levans [75]. These long-chain extracellularly produced polysaccharides have hypoglycemic activities [76], possibly explaining, at least in part, the antidiabetic properties of kefir.

The current meta-analysis had several important limitations. There were only a small number of eligible trials, which enrolled a small number of participants and were of relatively short duration. The included trials also varied in the probiotic cultures and the comparators used, such as conventional yogurt and milk, which might potentially have influenced glycemic outcomes. The majority of trials did not account for differences in lifestyle (physical activity, diet, sleep, smoking, etc.) and chronic disease burden, which may contribute to glycemic control. A small sample of our meta-analysis precluded assessment of dose-dependent nonlinear associations. None of the trials examined the composition of the gut microbiome at baseline and following probiotic administration to properly assess the impact of pre-existing flora on subsequent outcomes. In addition, residual unaccounted confounding might have impacted the outcomes. Finally, two of the trials included obese subjects without diabetes [33,34]. One of the two studies also included prediabetic subjects [33]. Overweight and obese individuals are at high risk of developing diabetes and impaired glycemic control. It is well known that the microbiome plays a role in obesity and hence the decision to include these trials [12,13,14]. Despite the importance of these studies in understanding the use of probiotics for metabolic diseases, we acknowledge that the microbiome may differ in these obese, nondiabetic patients. Notwithstanding these limitations, this meta-analysis had several strengths. This is the first meta-analysis, to our knowledge, evaluating probiotic yogurt contribution to glycemic outcomes. The data, including a trend for HbA1c reduction, could be interpreted as suggesting, though not proving, the glycemic benefits of probiotic yogurt. Analysis of probiotic yogurt per se compared with previous meta-analyses that included probiotic capsules was important. Probiotic capsule supplements can vary in probiotic content depending on the manufacturer, can be expensive, and their availability can be limited [77,78]. Probiotic yogurt, which has been recommended as an excellent source of protein and other nutrients, would be inexpensive compared to capsules. Probiotic yogurt is becoming widely available worldwide and could be included in recommendation of three units per day of dairy consumption.

## 5. Conclusions

The present meta-analysis has not demonstrated the benefits of consuming probiotic yogurt compared with conventional yogurt for improving glucose control in patients with type 2 diabetes or obesity. Larger randomized trials are needed to provide proof of principle and determine the practical impact of probiotic and/or conventional yogurt or kefir on glycemic markers in patients with diabetes and obesity. It is preferable that any future studies last 12 weeks or longer to fully assess an impact of probiotic supplementation on hemoglobin A1c. Future studies may also examine the microbiome before and after treatment with probiotic supplements to assess beneficial changes in gut flora.

## Figures and Tables

**Table 1:** Characteristics of included trials and data for participants at baseline.

Author Country Year	Design Duration	Subject Number T/C, Male/Female	Age BMI T/C	Treatment, Dose	Control, Dose	Probiotic Strains and Strength (CFU/mL)	Glycemic Out-Comes
T2D							
Bayat, Iran, 2016 [24]	Open-label, RP 8 wks	20/20 12/28	54/47 29/30	PY 150 g/d	Dietary advice	Cultures not provided	A1c, FBG
Ejtahed, Iran, 2012 [25]	DB, PC, RP 6 wks	30/30 23/37	51/51 29/29	PY 300 g/d	CY 300 g/d	*L. acidophilus* La5 and *B. lactis* Bb12 (1.8 × 10^6^ both)	A1c, FBG, insulin
Hove, Denmark, 2015 [26]	DB, PC, RP 12 wks	23/18 41/0	59/61 29/28	PY (low fat) (Cardi04^®^ yogurt) 300 mL/d	Milk (low fat) (Artificially acidified milk) 300 mL/d	*L. helveticus* (Cardi04) (CFU not provided)	A1c, FBG, insulin, HOMA-IR, C-peptide, proinsulin
Mohamadshahi, Iran, 2014 [27]	DB, PC, RP 8 wks	21/21 30/32	53/49 28/29	PY 300 g/day	CY 300 g/day	*L. acidophilus* La-5 and *B. lactis* BB-12 (10^6^ both)	A1c, FBG
Moroti, Brazil, 2012 [28]	DB, PC, RP 30 days	9/9 0/18	56/57 28/28	PY (Symbiotic shake) 100 mL BID	CY (shake without active ingredients) 100 mL BID	*L. acidophilus*, *B. bifidum* (4 × 10^8^ both) and 1 g oligofructose	FBG
Ostadrahimi, Iran, 2015 [29]	DB, PC, RP 8 wks	30/30 34/26	35-65 29/27	PY (kefir, low fat) 600 mL BID	CY (dough, low fat) 600 mL BID	*L. casei*, *L. acidophilus*, *B. lactis* (10^6^ both)	A1c, FBG
Tonucci, Brazil, 2017 [30]	DB, PC RP 6 wks	23/22 26/19	52/51 28/28	PY (goat milk) 120 g/d	Milk (goat milk) 120 g/d	*L. acidophilus* La-5 (6 × 10^7^), B. lactis BB-12 (2 × 10^7^)	A1c, FBG, insulin, HOMA-IR, fructosamine
OW/OB							
Ivey, Australia, 2014 [33] ^a^	DB, PC, RP 6 wks	37/40 48/29	68/65 30/31	PY 200 mL/d	Milk 200 mL/d	*L. acidophilus* La-5, B. lactis Bb-12 (3 × 10^9^)	A1c, FBG, insulin, HOMA-IR
Madjd, Iran, 2016 [34]	SB, PC, RP 12 wks	44/45 0/89	32/32 32/32	PY (low fat) 200 g BID	CY (low fat) 200 g BID	*L. acidophilus* LA-5, B. lactis BB12 (10^7^ both)	A1c, FBG, 2-h PG, insulin, HOMA-IR

^a^ Ivey trial included two additional groups using probiotic capsules; these groups were not included in the meta-analysis. Abbreviations: 2-h PG, 2-h postprandial glucose; A1c, hemoglobin A1c; B, *Bifidobacterium*; BID, twice a day; BMI, body mass index; FBG, fasting blood glucose; C, control; CO, cross-over; CY, conventional yogurt; d, day; DB, double-blind; L, *Lactobacillus*; OB, obese; OW, overweight; PC, placebo-controlled; PY, probiotic yogurt; RP, randomized-parallel groups; SB, single-blind; T, treatment; T2D, type 2 diabetes; wks, weeks.

**Table 2:** Glycemic outcomes in diabetes and obesity trials.

Glycemic Outcome	Baseline Data		Final Data		*p*-Value ^a^
	Control	Treatment	Control	Treatment	
**HbA1c, %**					
**T2D subjects**					
Bayat	7.54 [2.03]	7.06 [1.58]	7.55 [1.87]	6.49 [1.33]	<0.01 ^c^
Ejtahed	6.87 [0.81]	7.29 [1.21]	7.17 [0.66]	7.17 [1.24]	<0.05 ^d^
Hove	7.3 [0.6]	6.8 [0.5]	7.7 [0.9]	7.1 [0.6]	NS
Mohamadshahi	8.33 [1.46]	8.24 [1.68]	8.09 [1.58]	7.09 [1.23]	<0.05 ^c,d^
Ostadrahimi	6.98 [1.63]	7.61 [1.22]	7.0 [1.98]	6.40 [1.91]	0.02 ^d^
Tonucci	5.82 [1.04]	6.38 [1.04] ^e^	5.94 [1.02]	6.09 [1.17]	0.02 ^b^
**Obese subjects**					
Ivey	5.56 [0.36]	5.86 [0.65]	5.60 [0.34]	5.74 [3.19]	NS
Madjd	5.01 [0.57]	5.11 [0.48]	4.74 [0.50]	4.81 [0.47]	NS
**FBG, mg/dL**					
**T2D subjects**					
Bayat	145.2 [41.9]	148.95 [47.26]	165.5 [41.34]	126.25 [34.01]	<0.01 ^c^
Ejtahed	132.3 [23.04]	145.08 [44.82]	135.54 [23.76]	132.48 [43.38]	<0.05 ^d^
Mohamadshahi	187.42 [55.13]	175.24 [46.63	185.19 [63.60]	157.28 [43.63]	NS
Moroti	136.78 [19.47]	191.11 [18.31]	110.56 [29.90]	116.78 [18.96]	<0.05 ^b^
Ostadrahimi	183.42 [74.76]	161.63 [57.71]	182.16 [73.78]	139.22 [182.16]	0.02 ^d^
Tonucci	132.8 [42.7]	142.0 [41.0]	135.7 [45.0]	151.4 [43.4]	NS
**Obese subjects**					
Ivey	96.48 [9.9]	101.52 [18.18]	93.24 [11.7]	98.46 [13.14]	NS
Madjd	90.72 [8.28]	91.44 [8.64]	86.40 [7.74]	86.04 [7.92]	0.059 ^d^
**Insulin, μU/mL**					
**T2D subjects**					
Ejtahed	6.31 [3.72]	7.47 [4.89]	6.50 [3.57]	6.97 [4.49]	NS
Tonucci	7.90 [2.51]	8.53 [3.74]	7.84 [2.99]	8.14 [3.21]	NS
**Obese subjects**					
Ivey	9.99 [4.49]	9.63 [4.82]	10.18 [5.36]	10.59 [6.74]	NS
Madjd	12.69 [3.63]	12.85 [3.98]	11.36 [3.25]	11.09 [3.31]	<0.01 ^d^
**HOMA-IR**					
**T2D subjects**					
Tonucci	2.67 [1.17]	3.01 [1.41]	2.80 [1.38]	3.04 [1.31]	NS
**Obese subjects**					
Madjd	2.86 [0.92]	2.93 [1.03]	2.43 [0.77]	2.38 [0.80]	<0.01 ^d^
Ivey	2.44 [1.29]	2.48 [1.45]	2.38 [1.39]	2.63 [1.91]	NS

Data are mean [SD]. NS—not significant. ^a^ *p* for control vs. treatment at final visit; ^b^ difference between changes (final minus baseline); ^c^ unadjusted *p*; ^d^ *p* adjusted for baseline; ^e^ *p* < 0.05 for difference at baseline (treatment vs. control).
